# The effect of *Berchemia discolor* leaf meal (Muni tree) on gastrointestinal nematode infections of Creole goats

**DOI:** 10.5455/javar.2026.m1061

**Published:** 2026-06-22

**Authors:** Hypatia Thobejane, Tlou Grace Manyelo, Busisiwe Gunya

**Affiliations:** 1 Department of Agricultural Economics and Animal Production, University of Limpopo, Sovenga, Limpopo Province, South Africa

**Keywords:** *Berchemia discolor*, anthelmintic efficacy, gastrointestinal nematodes, oocyst count reduction

## Abstract

**Objectives:** This study evaluated the effect of *Berchemia discolor* leaf meal (Muni tree) on gastrointestinal nematode infections in Creole goats.

**Materials and Methods:** A total of 16 yearling intact males of Creole goats (12 months old, body weight 19.63 ± 1.68 kg) were randomly allocated to four dietary treatments in a completely randomized design. Experimental diets contained 0%, 15%, 20%, and 30% *B. discolor* leaf meal, formulated on a dry matter basis for 42 days. Fecal samples were freshly collected directly from the rectum on the last day using a modified McMaster technique to determine fecal egg counts (FEC) and fecal oocyst count (FOC). Data was analyzed using one-way ANOVA in SPSS version 30.0.

**Results:** Supplementing Creole goats with *B. discolor* influenced *Strongyloides* (*p* < 0.05). No *Strongyloides* eggs were detected in *B. discolor* at 20% or 0%, while higher counts were observed in *B. discolor* at 30% and 15%. Significant differences (*p* < 0.05) were also observed in *Trichuris* sp. and oocyst counts among dietary treatments. Correlation analysis indicated a positive relationship between *B. discolor* inclusion level and *Strongyloides.* counts, whereas a negative relationship was observed for *Trichuris* sp. and oocyst counts.

**Conclusions:** The findings suggest that *B. discolor* has the potential as a natural feed supplement for the management of gastrointestinal nematode infections in goat production systems.

## 1. Introduction

Parasites significantly hinder goat production by increasing mortality rates, causing skin irritation and blood loss, and reducing fertility [1, 2]. Among the primary health concerns in small ruminants are gastrointestinal parasites such as *Haemonchus contortus* and *Fasciola hepatica.* According to Mekonnen et al. [3], ectoparasites such as ticks, lice, and mites are prevalent among goats and contribute to health complications. In developing countries, the high prevalence of parasitic infections is attributed largely to inadequate veterinary services, poor housing, and limited access to feed resources [4].

Commercial anthelmintics are available for parasite control, but they are often expensive and hence inaccessible to smallholder farmers [5-7]. In addition, many parasites have developed resistance to one or more drug classes, reducing efficacy and increasing risk of environmental contamination through overuse [5, 7]. Therefore, natural remedies, particularly the use of tannin-rich forages or medical plants, have been studied to be the alternative use for farmers to control and treat parasitic livestock. These natural remedies are considered environmentally friendly and compatible with organic farming systems [8]. *Berchemia discolor*, also known as the Muni tree, which is a forage species beneficial to goat health, is one of these natural plant remedies. The presence of tannin-rich plants in forested areas has been associated with reduced worm burdens and improved goat performance [9]. *Berchemia discolor* is noted for its high tannin content, which plays a role in the management of parasitic infections among livestock by disrupting the development and survival of nematodes [10].

A total of 16 yearling intact males of Creole goats (averaging 12 months old, body weight 19.63 ± 1.68 kg) were randomly assigned to four dietary treatments in a completely randomized design and fed experimental diets containing 0%, 15%, 20%, and 30% *B. discolor* leaf meal, formulated on a dry matter basis with a mixture of pellets and lucerne hay for 42 days. However, notwithstanding the ethnobotanical relevance of *B. discolor*, there is a notable lack of empirical data evaluating its efficacy as a feed supplement specifically targeting gastrointestinal nematode (GIN) infections in goats under controlled experimental conditions. Therefore, the objective of the study was to determine the effect of *B. discolor* leaf meal (Muni tree) on gastrointestinal nematode infections of Creole goats.

## 2. Materials and Methods

### 2.1. Ethical statement

All animal handling procedures were approved by the Animal Research and Ethics Committee (AREC) of the University of Limpopo with the AREC number AREC/26/24: PG.

### 2.2. Study site

The study was conducted at the Animal Unit of the University of Limpopo, South Africa (1282 m altitude, 27.55°S latitude, and 29.41°E longitude). The study area experiences summer temperatures between 20 and 36°C and winter temperatures between 10 and 25°C. It averages lower than 400 mm of precipitation annually [11].

### 2.3. Acquisition of materials and goats

**Figure 1.**
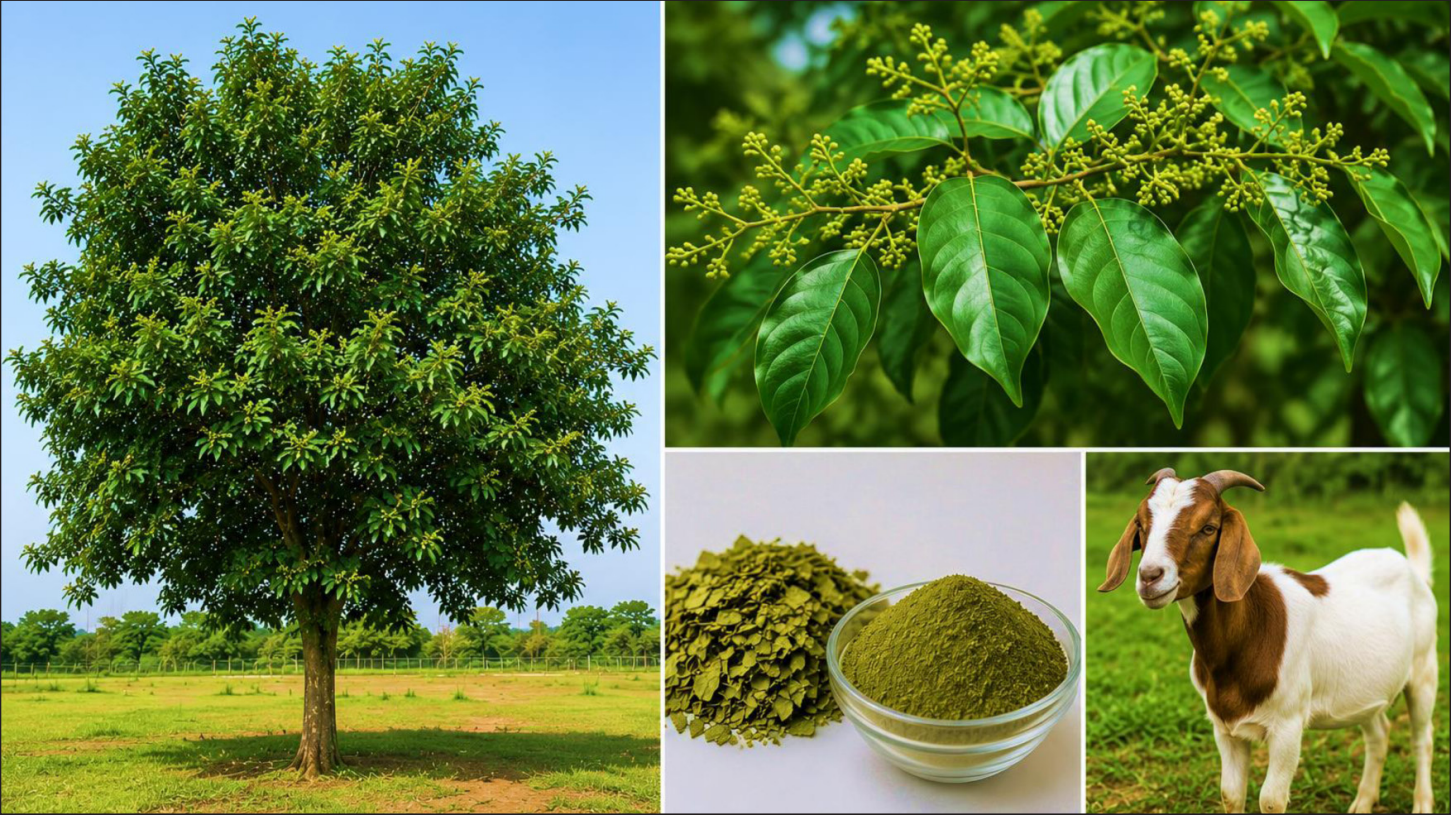
*Berchemia discolor* (Muni tree), its leaves, and leaf meal used in the study; and a representative Creole goat.

A total of 16 yearling intact males of Creole goats (averaging 12 months old and with a body weight of 19.63 ± 1.68 kg) were acquired from the University of Limpopo’s animal farm. *Berchemia discolor* leaf material was manually harvested from Hamakuya Village, Thohoyandou (latitude –22.64°E and longitude 30.84°S) (Figure 1). The leaves from the branches were then shade-dried and kept indoors for 16 days before being chopped. The samples were cut and kept in sealed bags until they were required for analysis and feeding.

### 2.4. Experimental procedures, dietary treatments, and housing

#### 2.4.1. Experimental house procedure, goats, and feeding management

The experimental individual metabolic cages were carefully cleaned with water and a sterilizer (ViruKill) and then left vacant for 7 days to allow for adequate drying and the death of disease-causing microorganisms. The metabolic cage was separated into three equal-sized floors, with fresh sawdust spread out to a 7 cm depth. Yearling intact males of Creole goats were housed in a 1.5 m × 0.8 m individual pen of the specified dimension and acclimated to the trial food for 14 days. There was a total of 16 pens. The feeding trial was for 42 days, with a 7-day adaptation period. 600 gm of supplements were given to goats two times a day (10:00 AM and 4:00 PM). Goats were fed individually, and water troughs with a basic meal and unlimited access to water were provided.

#### 2.4.2. Experimental design and treatments

The experiment followed a complete randomized design (CRD) with four dietary treatments and four replicates per treatment (*n* = 16 goats). Each goat served as an experimental unit. The dietary treatments were as follows: B₀: Control, B₁₅: 15% inclusion of *B. discolor* leaf, B₂₀: 20% inclusion of *B. discolor* leaf, and B₃₀: 30% inclusion of *B. discolor* (BFL₀, BFL₁₅, BFL₂₀, and BFL₃₀) is shown in Table 1, Composition of feed materials in the experimental diets. Each goat received 600 gm of the respective diet per feeding, twice daily (total 1.2 kg/day).

**Table 1.** Composition of feed materials in the experimental diets.

Feed	Co	BFL_15_	BFL_20_	BFL_30_
*Berchemia discolor* leaf (%)	0	15	20	30
Hay (*Cenchrus ciliaris*) (%)	66	51	46	36
Lucerne pellets (%)	34	34	34	34
Total (%)	100	100	100	100

#### 2.4.3. Management

A total of 16 yearling intact male Creole goats were acclimated to an experimental diet for 14 days in an individual pen with 1.5 m long × 0.8 m wide dimensions to allow some levels of interaction between animals to avoid stress associated with isolation. Random sampling was carried out from the intact yearling Creole goats during feces sample collection to avoid disturbing their normal routine. Goats were gently guided following animal handling procedures [12] to minimize stress and discomfort and to decrease the risk to animal health. Health assessment and monitoring of the goats were carried out by a veterinarian. Loud noise, sudden movements, or crowded conditions that could make the goat anxious were avoided. At the end of the study, goats were returned to the university farm. 

#### 2.4.4. Feces collection

Fresh fecal samples were collected directly from the rectum of each goat on the final day of the experiment; soil or bedding contamination was avoided. Samples were placed into labeled containers (animal ID and date) and promptly analyzed for fecal egg count (FEC) and fecal oocyst counts (FOC). Samples were refrigerated (4°C) and analyzed within 12-24 h since, once hatched, they cannot be counted. The modified McMaster technique was used. Fecal egg count was used to measure how many parasite eggs a goat is passing in each gram, while fecal oocyst counts measured the number of protozoan oocysts in each gram of feces.

### 2.5. Statistical analysis

The data for feces samples was analyzed with a one-way analysis of variance (ANOVA) using a statistical software program, the Statistical Package for the Social Sciences (SPSS), version 30.0. Treatment means we were separated using Duncan’s post hoc test at a significance level of *p* < 0.05. Results are presented as the mean ± the mean of the standard error (MSE). Correlation coefficients (*r*) were calculated to assess the relationship between *B. discolor* inclusion levels and parasite load indicators. Regression analysis confirmed a negative linear relationship between *B. discolor* inclusion and FEC (R^2^ = 0.42, *p* < 0.05).

## 3. Results

Table 2 showed the effects of *Berchemia discolor* leaf meal supplementation on gastrointestinal nematode infections of Creole goats. Supplementation with *B. discolor* significantly influenced (*p* < 0.05) the prevalence of *Strongyloides*. Eggs per gram and oocyst per gram values present the mean parasite load per group of animals. The parasite load comparison was made only between dietary treatments to evaluate the effect of increasing *B. discolor* inclusion levels on gastrointestinal nematode infection intensity. Different superscripts (^a, b, c, d^) within a column indicate statistically significant differences among treatments (*p* < 0.05).

**Table 2.** Fecal Sample Analysis for Nematode Egg Count and Oocyst Counts.

Treatment	*Strongyloides*	*Trichuris* sp.	Oocyst
BFL_0_	0.00 ± 0.0^a^	1210.0 ± 11.55^b^	3427.5 ± 3.75^c^
BFL_15_	302.5 ± 2.88^b^	650.0 ± 57.74^a^	10850.0 ± 57.74^c^
BFL_20_	0.0 ± 0.0^a^	1125.0 ± 28.87^b^	594.0 ± 51.96^a^
BFL_30_	305.0 ± 5.77^b^	930.0 ± 25.55^a^	2610.0 ± 11.55^b^

BFL = *Berchemia discolor* feed level. _0, 15, 20, 30_: different levels of *Berchemia discolor* feed. Superscripts (^a, b, c, d^) indicate statistical significance. Means with different superscripts differ significantly at *p* < 0.05.

The highest counts were recorded in goats fed BFL_3.0_, followed by BFL_1.5_, whereas no *Strongyloides* eggs were detected in BFL_0_ and BFL_2.0_ groups. A significant (*p* < 0.05) difference was observed for *Trichuris* sp. infection. The highest *Trichuris* sp. counts are in BFL_0_, followed by BFL_2.0_, BFL_3.0_, and BFL_1.5_ oocyst counts. However, there was no significant difference between BFL_0_ and BFL_2.0_ or BFL_1.5_ and BFL_3.0_. *Berchemia discolor* had an influence (*p* < 0.05) on oocyst infection among the dietary treatments. The highest oocyst value was observed on BFL_1.5_, followed by BFL_0_, followed by BFL_3.0_, and the smallest values were recorded on BFL_2.0_.

Correlation analysis revealed a positive relationship between *B. discolor* inclusion level and *Strongyloides* count (r² = 0.337) of Creole goats, whereas negative relationships were observed between *B. discolor* leaf meal and *Trichuris* sp. (r² = 0.139) and oocyst (r² = 0.33) of Creole goats. Relationship between *B. discolor* leaf meal, FEC (*Strongyloides* and* Trichuris* sp.), and FOC (oocyst) (Table 3). Y = predicted response variable (eggs per gram or oocyst per gram); X = *B. discolor* inclusion level (%); r² = coefficient of determination showing the proportion of variation in parasite load explained by *B.* *discolor* level.

**Table 3.** Relationship between *Berchemia discolor* leaf meal and FEC (*Strongyloides* and *Trichuris* sp.) and FOC (oocyst).

Variable	Formula	*R* ^2^	Probability
*Strongyloides*	Y = 8.14x + 19,6	rᵌ = 0.037	0.042
*Trichuris* sp.	Y = –7.44x + 1099	rᵌ = 0.139	0.628
Oocyst	Y = 63.80x + 5489.65	rᵌ = 0.33	0.818

Y, linear regression formula. r^3^, correlation coefficient.

The regression analyses show a weak and statistically non-significant relationship (*p* > 0.05) between *B. discolor* supplementation and the counts of *Trichuris* sp. and oocysts, while a marginally significant association (*p* = 0.042) was observed for Strongyloides, suggesting a potential dose-related response. Y = predicted response variable (EPG or OPG); X = *B. discolor* inclusion level (%); r² = coefficient of determination showing the proportion of variation in parasite load explained by *B. discolor* level.

The regression analyses show a weak and statistically non-significant relationship (*p* > 0.05) between *B. discolor* supplementation and the counts of *Trichuris* sp. and oocysts, while a marginally significant association (*p* = 0.042) was observed for *Strongyloides*, suggesting a potential dose-related response. Although correlations between *B. discolor* inclusion and parasite reduction were weak (r² = 0.4), the consistent downward trend suggests a moderate biological relevance that warrants further study with larger samples. 

## 4. Discussion

One of the primary factors limiting ruminant output that impacts animal performance is infestation by internal parasites; gastrointestinal nematodes become more resilient and resistant when the amount of digestible protein increases [13]. Supplementation with* Berchemia discolor* had a slight effect on FEC and worm burdens. This finding is consistent with previous studies [14, 15, 16, 17], which reported lower effects on parasite-infested sheep or goats that were offered tanniniferous fodder.

In this study before goats were fed, *B. discolor* had high FEC; to ascertain that, goats in BFL_0_ were not given *B. discolor,* and all the BFLs that were given *B. discolor* had lower infestation compared to BFL_0_. According to Max et al. [18], the provision of quebracho tannins as a drench to sheep had caused a significant reduction in their worm burden; these findings are similar to this study’s findings: plant leaf meal had an effect on worm reduction.

*B. discolor* influenced FEC and significantly reduced their worm burden. This contradicts the finding by Rodríguez-Hernández et al. [19], who reported that there was no significant effect observed on* Quebracho tannins* against the FEC. This deviation might be because of the use of different leaf meals, as *B. discolor* and quebracho contain structurally distinct condensed tannins with varying biological activity. In the current study, we used *B. discolor* and influenced FEC and significantly reduced their worm burden. Differences among studies may arise from variation in tannin concentration, chemical structure, animal species, and parasite populations [19].

The fecal egg count of the infected goats treated with *B. discolor* significantly decreased over the 28-day course of BFLs in heavy infection with the complete disappearance of eggs in light infection. This occurred because *B. discolor* supplementation decreased fecal egg production, which was followed by decreased egg hatchability and larval development, which then resulted in a decrease in infestation. Similar reductions in egg hatchability and larval development have been reported for several tannin-containing plants used in parasite control programs [20]. These findings are consistent with previous research by Hegazi et al. [21], which demonstrated the ovicidal action of *M. oleifera* leaf extracts, both alcoholic and aqueous, against Fasciola eggs *in vitro*. Findings of this study differ from the finding by Ramírez-Restrepo et al. [22], who reported that worm fecundity was negatively impacted by acacia supplements. This deviation might be because of the use of different leaf meals. In the current study, we used *B. discolor*, and Ramírez-Restrepo et al. [22] used *acacia* supplements.

In this study,* B. discolor* influenced the FOC. Coccidia was present in every goat used in this investigation. These findings agree with those of Clark et al. [23], who reported that* Mucuna pruriens* (MUC) did not reduce FEC or FOC*.* In this study, *B. discolor* leaf meal did not reduce oocyst values. These findings are in contrast with Hoste et al. [24], who reported that although *Gliricidia sepium* leaves can lessen moderate-to-low worm infestation levels, they are not as successful at eliminating worms as chemical medications like albendazole. Leaves from *G. sepium* can be used to manage worm infestations in the sense that sheep that have severe worm infestations need to be dewormed at the right time. The differentiation between these might be because different plant meals were used, which are *B. discolor* and *G. sepium*.

According to this study, goats on BFLₒ were not fed *B. discolor*, and other remaining BFLs were fed *B. discolor* and had high FOC compared to BFLₒ. This contradicts Lin et al. [25], which reported that FOC in goats was reduced by feeding leucaena (*Leucaena leucocephala*). This deviation could be because two distinct plant meals were used, *B. discolor* and *G. sepium.* In this research study BFLₒ was the only BFL that was not fed *B. discolor* and, as a result, had lower FOC values.

In this study, both low (15%) and high (30%) inclusion levels of *B. discolor* leaf meal were more effective in reducing FEC and worm burden than the medium (20%) level. This could be attributed to the dose-dependent response of condensed tannins. At lower concentrations (15%), tannins may enhance protein binding and reduce nutrient availability for parasites without impairing host digestion. At a higher inclusion level (30%), the stronger presence of tannins likely exerts direct toxic effects on nematode larvae and eggs, leading to pronounced reductions in FEC. However, at intermediate levels (20%), tannins may be insufficiently concentrated to cause parasite mortality while still moderately binding dietary proteins, thus diminishing their bioefficacy.

## 5. Conclusions

Supplementation of *Berchemia discolor* leaf meal significantly reduced gastrointestinal nematode infections of Creole goats. Goats that were fed *B. discolor* had lower fecal egg counts (FEC) and worm burdens than the control group (BFL_0_), with 15% (BFL₁.₅) and 30% (BFL₃._0_) inclusion levels showing the most pronounced reduction in *Trichuris* and *Strongyloides* infections. The antiparasitic effect is linked to condensed tannins that inhibit parasite development and reproduction. BFL_2.0_ supplementation also lowered coccidial oocysts, suggesting wide efficacy benefits. Overall, *B. discolor* leaf meal represents a promising, locally available, and sustainable alternative to synthetic anthelmintics. Its inclusion in goats’ diets could improve animal health, productivity, and resistance management for smallholder farmers where resources are limited.

## Data Availability

The data presented in this study are available from the corresponding author upon reasonable request.

## List of abbreviations

ANOVA, analysis of variance; AREC, Animal Research and Ethics Committee; CRD, complete randomized design; GIN, gastrointestinal nematode infections; FEC, fecal egg count; FOC, fecal oocyst count; MSE, mean of standard error; SPSS, Statistical Package for the Social Sciences; mm, millimeters; kg, kilograms; m, meter; gm, grams; cm, centimeters; h, hour.
